# Supplementary figures and images for: Blind estimation and correction of microarray batch effect
Source: PLoS One. 2020 Apr 9;15(4):e0231446. doi: 10.1371/journal.pone.0231446 (PMC7145015; doi:10.1371/journal.pone.0231446)

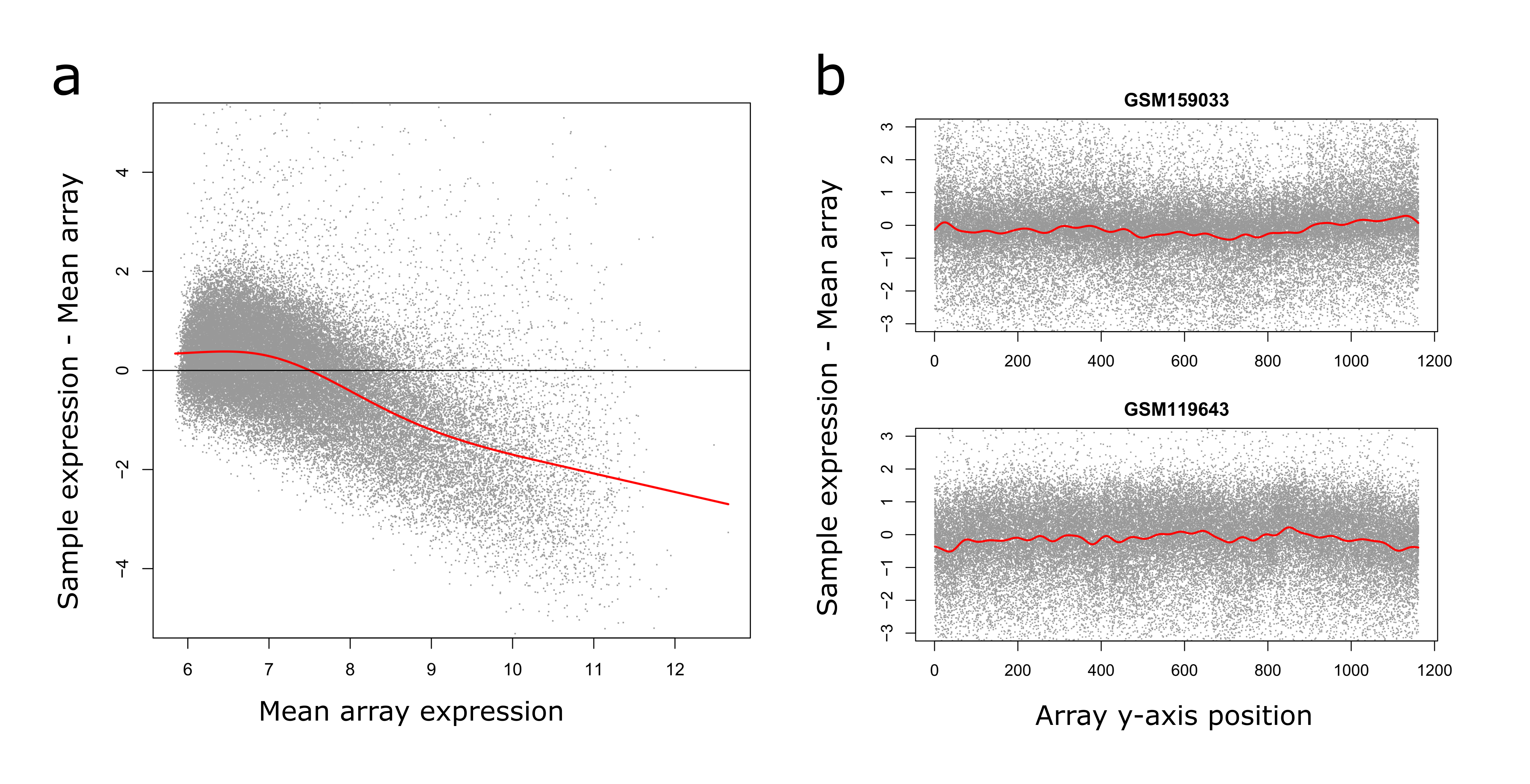

Supplement: S1 Fig — a) Bias in measured expression that depends on intensity b) Bias in measured expression that depends on array y-axis. (TIF) [file pone.0231446.s001.tif]

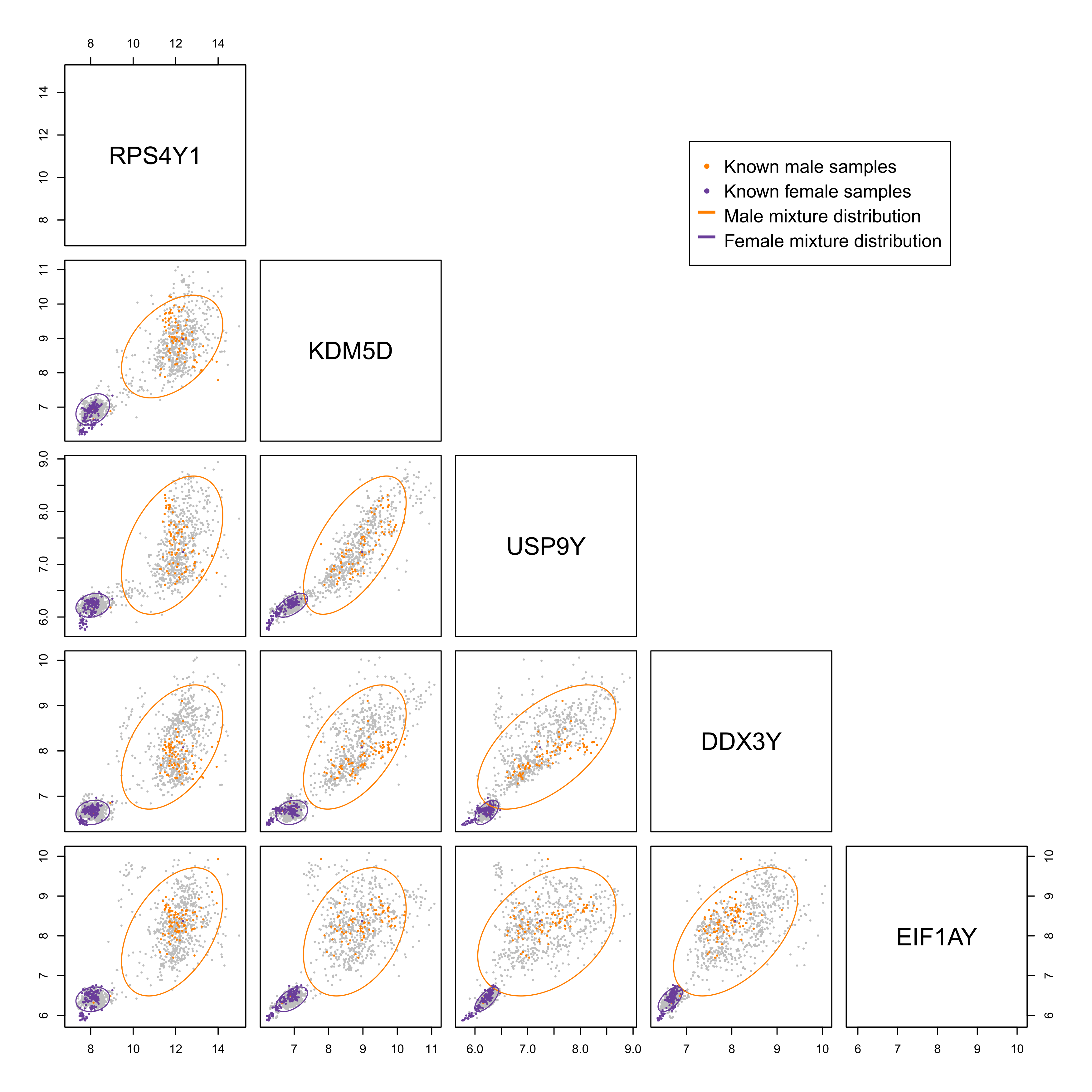

Supplement: S2 Fig — Scatterplots of sex related genes with known sex and ellipse of 95% density for fitted Gaussian mixture model. (TIF) [file pone.0231446.s002.tif]

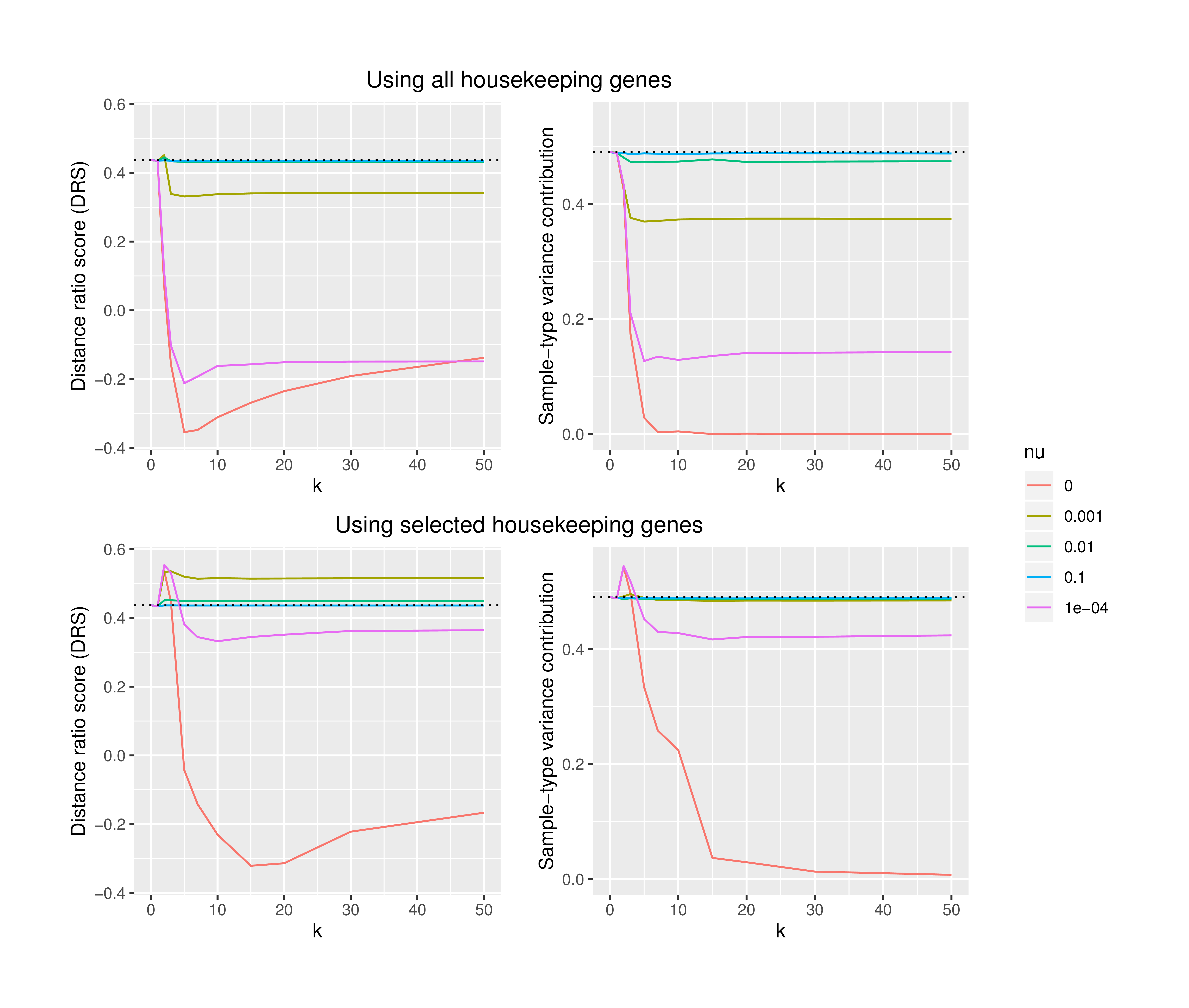

Supplement: S3 Fig — Plot of DRS and PVCA for various levels of the tuning parameter for RUV as well as two different sets of housekeeping genes. (TIFF) [file pone.0231446.s003.tiff]

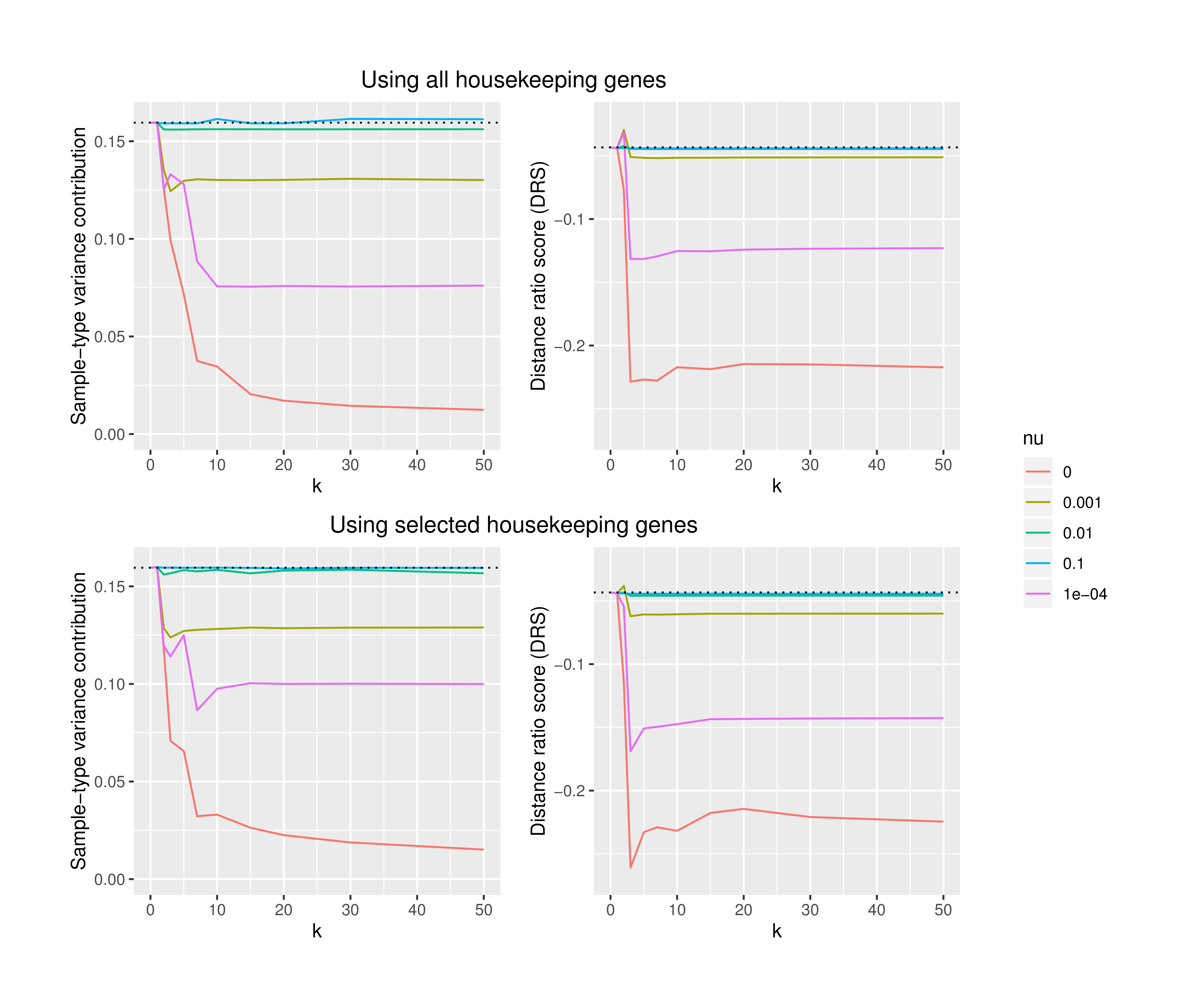

Supplement: S4 Fig — Plot of DRS and PVCA for various levels of the tuning parameter for RUV as well as two different sets of housekeeping genes. (TIFF) [file pone.0231446.s004.tiff]

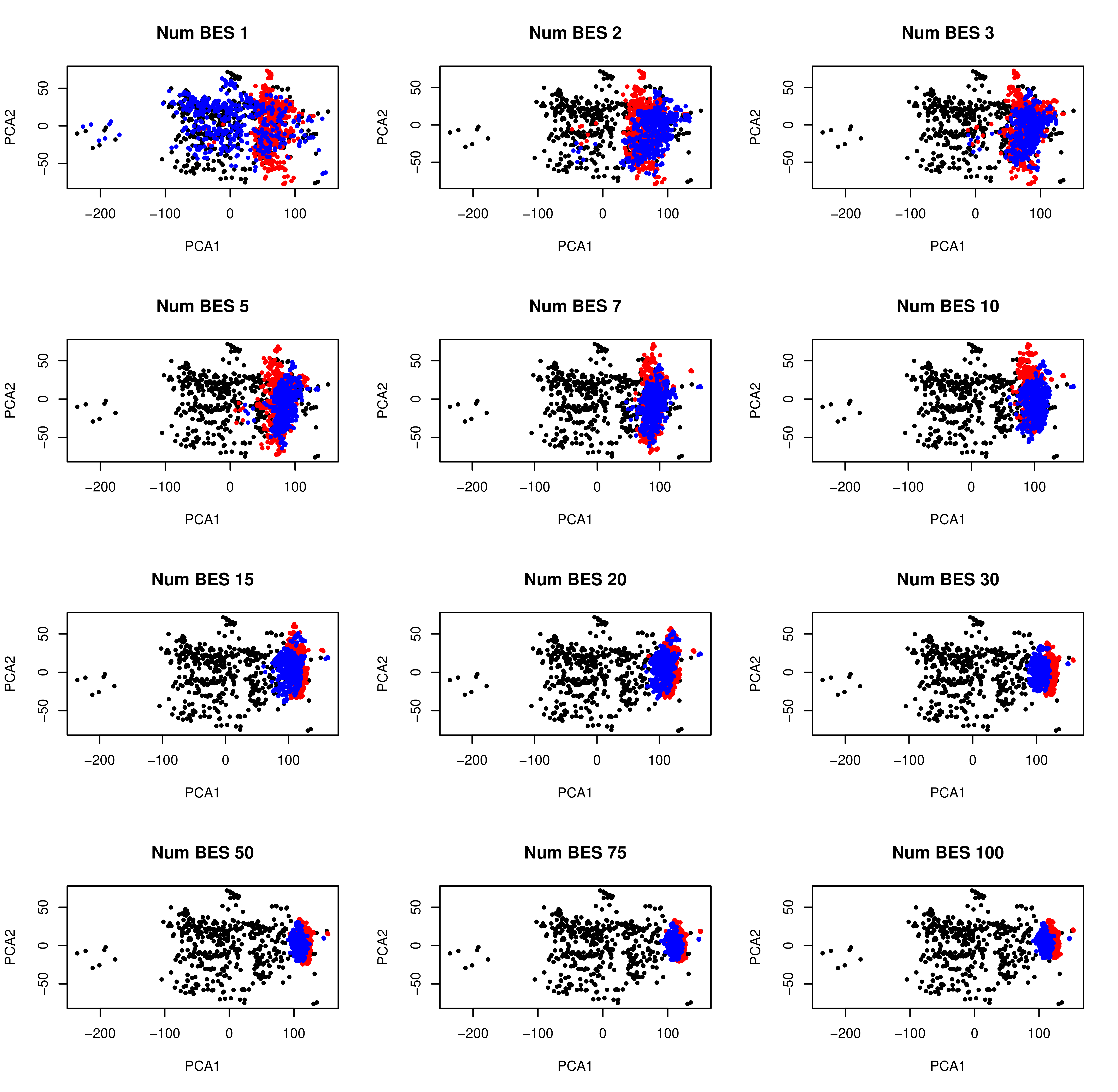

Supplement: S5 Fig — Overlap between significant genes selected for MSS/MSI differences in validation set 2 for BES calculated on different subsets of the reference set. (TIFF) [file pone.0231446.s005.tiff]
