# Supplementary material for: Blind estimation and correction of microarray batch effect
Source: PLoS One. 2020 Apr 9;15(4):e0231446. doi: 10.1371/journal.pone.0231446 (PMC7145015; doi:10.1371/journal.pone.0231446)
Supplement: S1 File — (DOCX) [file pone.0231446.s011.docx]

Supplementary methods

### Normalization of raw array data

Raw probe intensities were read from Affymetrix CEL files, log transformed and scaled to have the same mean and variance as the Mean-array of the Reference dataset samples. We found that there were two main sources of technical difference between arrays. The first was a nonlinear effect of the probe intensity on the difference with the Mean-array that showed as a “bending” up or down of the points in an MA plot (Fig 1a). The second was a dependence of probe intensity on the y-axis location of a probe that differed from array to array (Fig 1b).


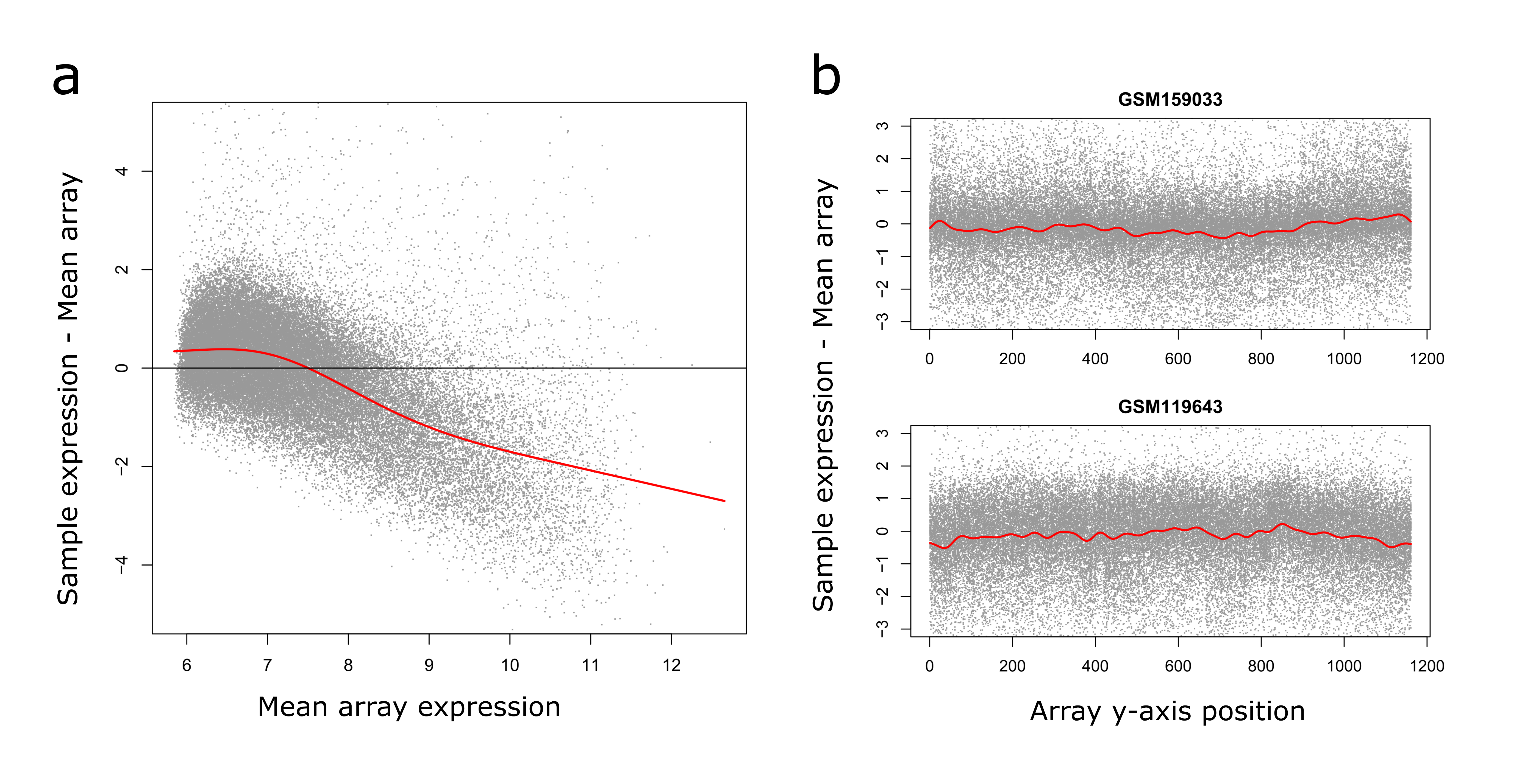


Figure 1 Normalization of arrays - a) Bias in measured expression that depends on intensity b) Bias in measured expression that depends on array y-axis

To remove both effects, we fitted a Generalized Additive Model (GAM) (Hastie and Tibshirani, 1990) using the smoothed values of the Mean-array intensity and the array y-axis positions to the difference between the array expression and the Mean-array. The array expression was corrected by subtracting the fitted values. The R package *mgcv* (Wood, 2011) was used for fitting the GAM.

### Summarization of expression for each gene

We used the Brainarray (Dai *et al.*, 2005) ENTREZG custom CDF (Version 18.0.0) for summarizing the batch corrected probe expressions. The custom CDF is a re-annotation of the probes on the Affymetrix U133 Plus2 array to pool together probes mapping to the same gene. Those probes were averaged together to estimate the expression of the gene.

### Prediction of sex for samples in the Validation set

To predict the sex of each sample in the Validation set, we used the expressions of five chrY genes (RPS4Y1, KDM5D, USP9Y, DDX3Y, EIF1AY). The sex of the subject was available for several of the Validation set samples from their GEO (Gene Expression Omnibus) annotations. We used the Student’s t-test on the known male and female samples (89 males, 143 females) to find chrY genes that were differentially expressed between the two sexes. The five selected genes had a p-value<1e-16 and greater than 2-fold change between males and females. Fig 2 shows the pairwise scatterplots for those five genes with the samples of known sex indicated.

We fitted a mixture of two multivariate Normal distributions on the expressions of those five genes using the R package *mixtools*. Samples with a-posteriori probability >0.5 of being male were classified as male and those with >0.5 probability of being female were classified as female. The ellipses in each panel in Supplementary Figure 2 show the region of expression containing 95% of the probability density for the fitted male and female distributions.


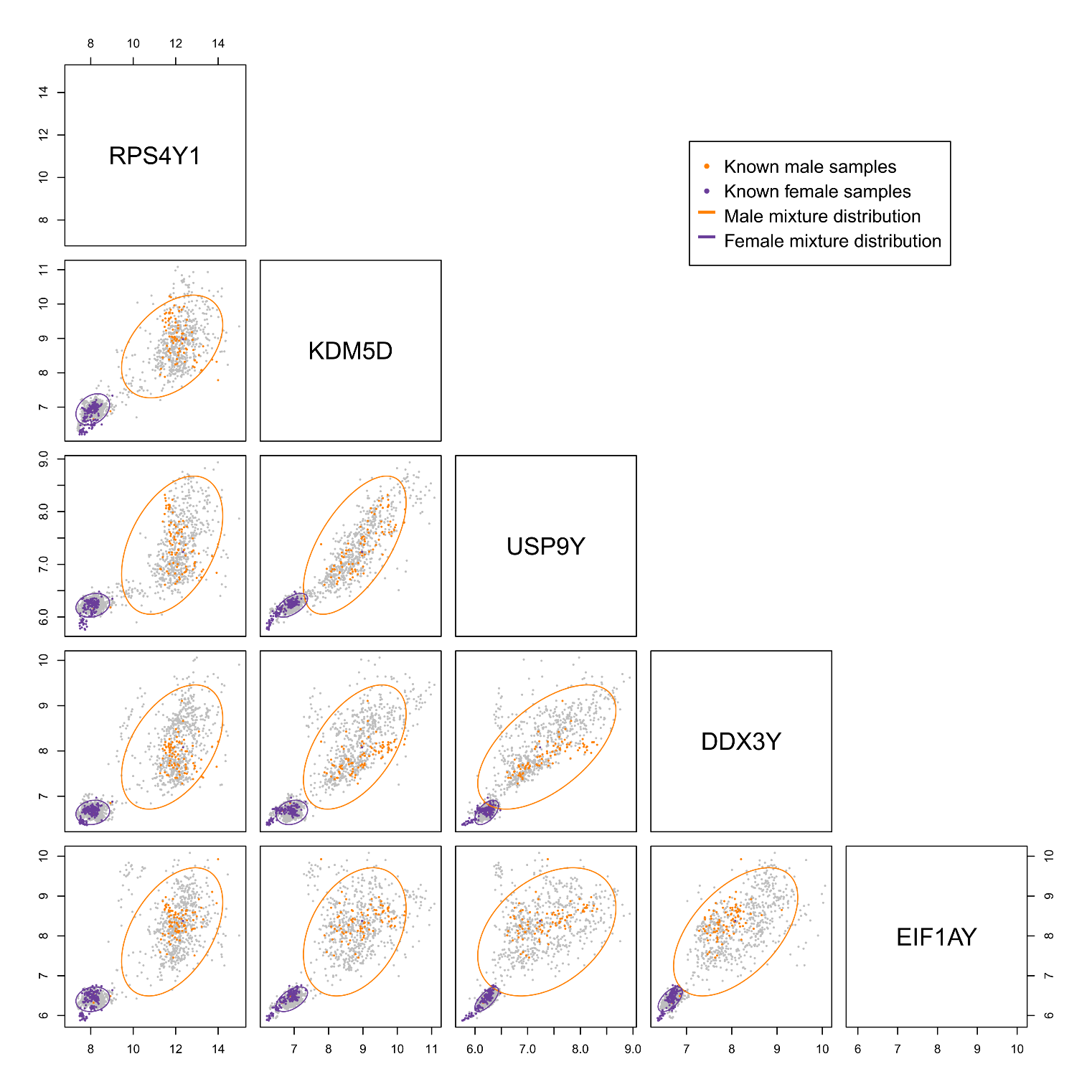


Figure 2: Prediction of sex - Scatterplots of sex related genes with known sex and ellipse of 95% density for fitted Gaussian mixture model

### Selection of housekeeping genes and $\boldsymbol{\nu}$ for *RUV*

We selected the set of housekeeping genes published in (Eisenberg and Levanon, 2003) (“Using all housekeeping genes in Figure 3 and 4). We found that a number of these genes were actually differentially expressed between sample groups (male *vs.* female in validation set 1 and microsatellite stable *vs.* unstable in validation set 2). Therefore we created another set of housekeeping genes by filtering out the genes from the first set that were differentially expressed between the sample groups (“Using selected housekeeping genes” in Figure 3 and 4)

###
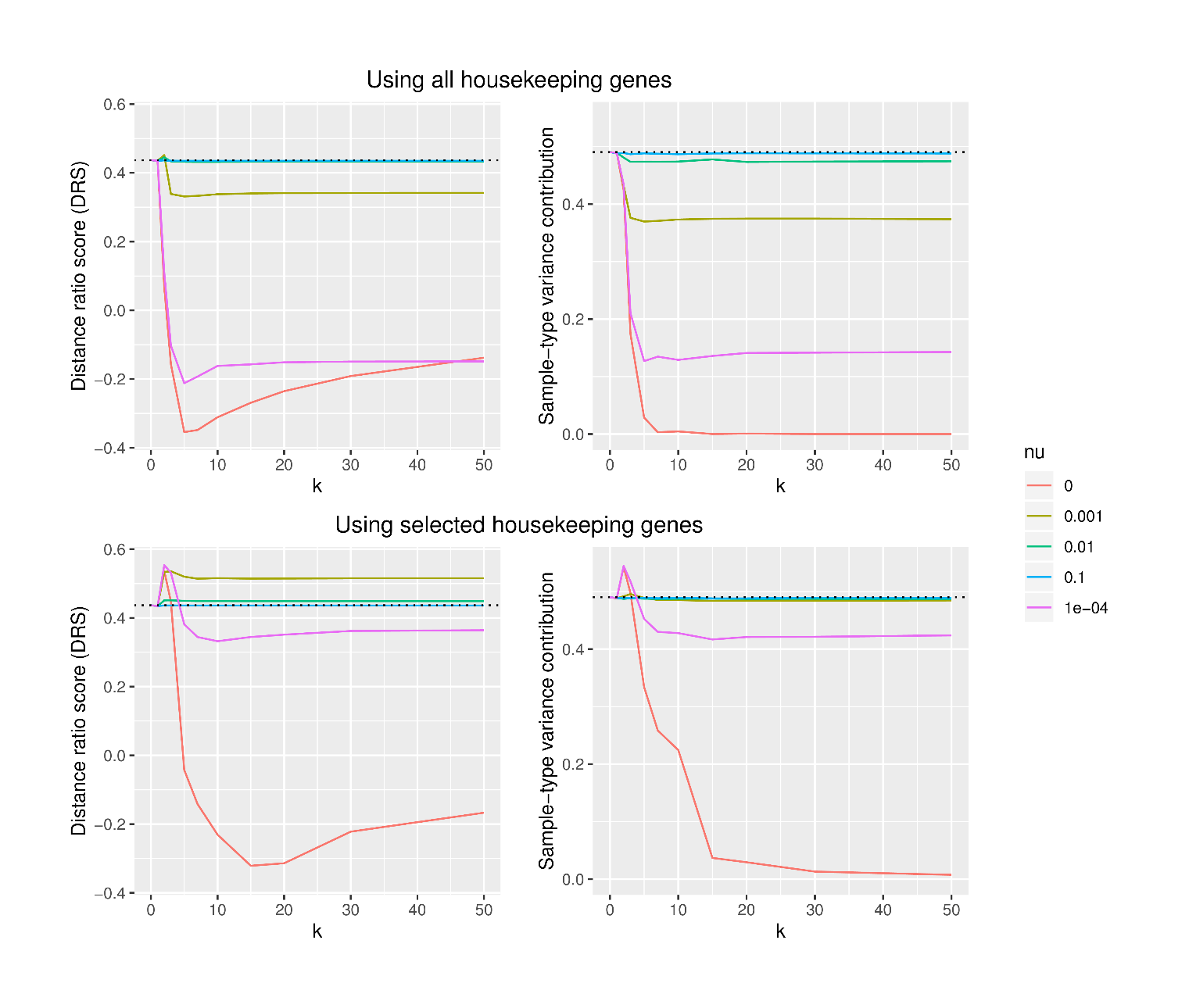


Figure 3: Selection of nu for the RUV algorithm for validation set 1

For validation set 1 (Figure 3), we selected a value of $\nu$=0.001 using the “selected housekeeping genes” (gold line in second row of Figure 3).

###
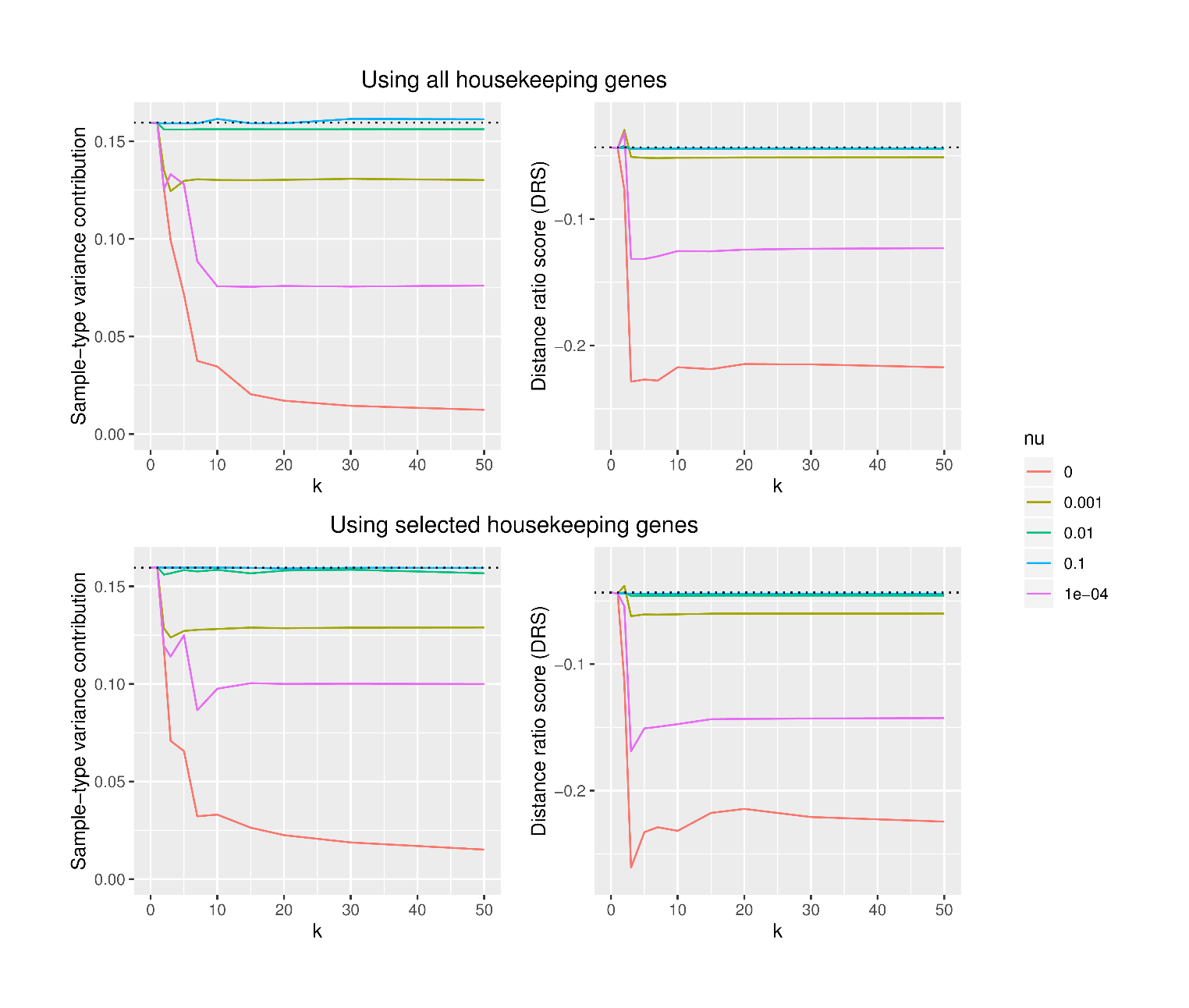


Figure 4: Selection of nu for the RUV algorithm for validation set 2

For validation set 2, we selected a value of $\nu$=0.1 with no control filtering (light blue line in top row).

### Comparison of BES calculated from different cross-validation splits

We looked at the splits created for the cross-validation and asked whether the BES calculated using samples from the different splits are similar (*i.e.* the *BESC* algorithm is picking up consistent signatures). For that, we combined splits 1 and 2 of the reference dataset into one dataset (“1+2”) and splits 3 and 4 into another dataset (“3+4”). Using these two datasets, we calculated two sets of BES (BES_1+2_ and BES_3+4_). Then we corrected split 5 as well as validation set 2 (colon) using these two sets of BES.

Figure 5 shows a PCA of the samples in split 5 with the original uncorrected (black) compared to the samples corrected by BES_1+2_ (red) and BES_3+4_ (blue). The two corrected datasets remain mostly together as the number of BES increases, indicating similarity of correction.

Figure 6 shows the mean correlation between the corrections performed by BES_1+2_ and BES_3+4_. We corrected a random vector using BES_1+2_ and BES_3+4_ and looked at the computed correction factors at different numbers of BES. The correlation between the correction factors increases with increasing number of BES, indicating that the two sets of BES compute similar correction factors even though they are calculated from different reference sets.

Together, these plots show that the corrections computed by subsets of the reference dataset are similar to each other, indicating a consistency in computing the BES.


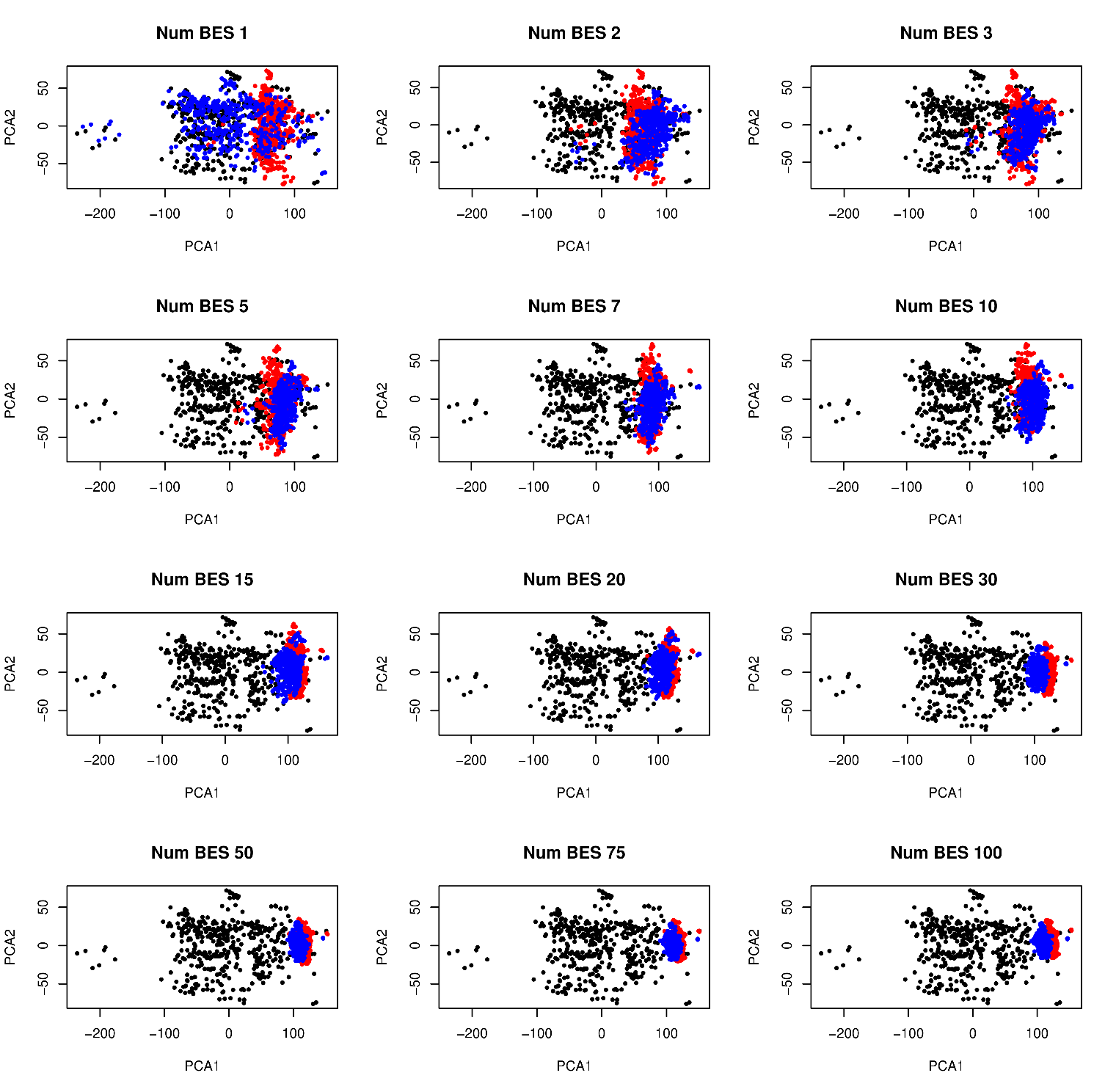


Figure 5: PCA of original samples (black points) from split 5 compared to those corrected by BES_1+2_ (red) and BES_3+4_ (blue) for increasing levels of correction





Figure 6: Average correlation between corrections done to a random vector using two sets of BES (BES calculated from splits 1+2 and BES from splits 3+4 of the reference set). The correlation increases till BES=10, indicating that the two sets of BES do similar corrections.


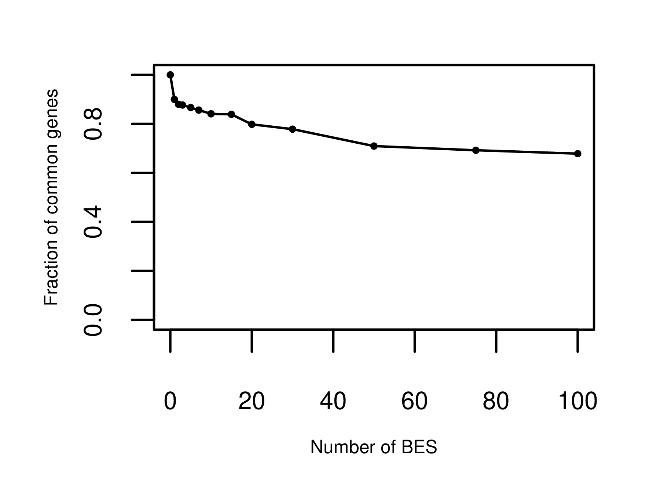


Figure 7: Percentage of overlap of MSS/MSI differential genes in the colon cancer dataset (validation set 2) between data corrected using two sets of BES (BES calculated from splits 1+2 and BES from splits 3+4 of the reference set). The percentage overlap is high (>80%) for the first 10 BES, indicating similar corrections using the two sets.

Figure 7 shows that there is a high level of commonality between the genes that are differentially expressed between MSS and MSI samples in validation set 2 when that dataset is corrected using BES_1+2_ and BES_3+4_. The intersection is more than 80% for all numbers of BES until 10, then decreases. That indicates that the correction done by the two sets of BES is similar as long as 10 or less BES are used.

### References

Dai,M. *et al.* (2005) Evolving gene/transcript definitions significantly alter the interpretation of GeneChip data. *Nucleic Acids Res.*, **33**, e175–e175.

Eisenberg,E. and Levanon,E.Y. (2003) Human housekeeping genes are compact. *Trends Genet. TIG*, **19**, 362–365.

Hastie,T. and Tibshirani,R. (1990) Generalized Additive Models CRC Press.

Wood,S.N. (2011) Fast stable restricted maximum likelihood and marginal likelihood estimation of semiparametric generalized linear models. *J. R. Stat. Soc. B*, **73**, 3–36.
