# Supplementary material for: Blind estimation and correction of microarray batch effect
Source: PLoS One. 2020 Apr 9;15(4):e0231446. doi: 10.1371/journal.pone.0231446 (PMC7145015; doi:10.1371/journal.pone.0231446)
Supplement: Attachment — Submitted filename: Response.docx [file pone.0231446.s013.docx]

1) The supplemental figures are not referenced in the main text. It is up to readers to detect that there are some supplemental figures and to try to figure out what they show. So please add some sentences in the main text and explain what the supplemental figures show, like you did in your reply to the reviewers.

I have added text to the manuscript describing those analyses and their results (lines 223-235 and 297-309)

2) Also, the legends of supplemental figures S5 to S7 should be improved. For example, the current legend of Fig. S5 "S5 Fig: Consistency of calculated BES – Overlap between significant genes selected for
   MSS/MSI differences in validation set 2 for BES calculated on different subsets of
   the reference set."does not reflect your reply to the comment of reviewer #1:
   "I’ve included a plot of the fraction of common genes between the colon cancer/normal
   dataset (validation set 2) when corrected by sets of BES calculated on the 1st + 2nd
   split combined and the 3rd + 4th split combined (Supplementary Figure 5).
   There is a high degree of overlap (>80% intersection) till the number of BES reaches 10.
   The BES start to diverge from 20 onwards and the overlap decreases."

   Similarly, the legends Figs. S6 and S7 could be improved.
   For example, the legend of Fig. S6 does not explain what the black, red and blue symbols are.
   I’ve update the legends and descriptions
3) The following small points refer to the line numbering of the revised manuscript with tracked changes
   a. line 108: the disadvantages ... is -> the disadvantages ... are
   b. line 387: correct plural/singular in "We show that the batch effect between different datasets occupy a space"

I have corrected these errors
